# Management of hypertension in chronic kidney disease: current perspectives and therapeutic strategies

**DOI:** 10.3389/fmed.2025.1630160

**Published:** 2025-10-29

**Authors:** Muath A. Alsalloum, Mohamed A. Albekery, Ibrahim S. Alhomoud

**Affiliations:** ^1^Department of Pharmacy Practice, College of Pharmacy, Qassim University, Qassim, Saudi Arabia; ^2^Department of Pharmacy Practice, College of Clinical Pharmacy, King Faisal University, Al-Ahsa, Saudi Arabia

**Keywords:** hypertension, chronic kidney disease, albuminuria, angiotensin-converting enzyme inhibitors, angiotensin receptor blockers, practice guideline

## Abstract

Hypertension in chronic kidney disease (CKD) is a major health challenge, with cardiovascular disease being the major cause of mortality in CKD. Several factors play a role in its pathophysiology, including renin-angiotensin system activation. Guidelines for blood pressure management in CKD patients demonstrate some variation in their recommended targets and therapeutic approach. However, current practice increasingly adopts stricter systolic blood pressure target when tolerable. A daily sodium intake of less than 2 grams and engagement in moderate-intensity physical activity (≥30 min, 5–7 days per week) are strongly recommended. However, the majority of patients with CKD ultimately require combination therapy with multiple antihypertensive agents, such as calcium channel blockers (CCBs) and thiazide or thiazide-like diuretics. Recent evidence is increasingly in favor of considering sodium-glucose cotransporter-2 (SGLT-2) inhibitors, incretin therapies, and mineralocorticoid receptor antagonists (MRAs), given their established benefits on cardiovascular and kidney-related outcomes, even though their blood pressure lowering effects remains relatively modest. Emerging agents with novel mechanisms of action, such as endothelin receptor antagonists, are also under investigation and may provide additional therapeutic options in the future. This review aims to summarize current guideline recommendations and therapeutic strategies for managing hypertension in CKD, including recent and emerging pharmacologic approaches.

## 1 Introduction

Hypertension in chronic kidney disease (CKD) is a major health challenge, with cardiovascular disease (CVD) being a major cause of morbidity and mortality in CKD. The prevalence of hypertension among CKD patients is higher than those without CKD, significantly increasing with the severity of CKD (1). Its overall prevalence is estimated at 80–85% (2). hypertension in CKD can lead to cardiovascular complications such as myocardial infarction (MI), heart failure, as well as stroke. This results in significant morbidity and mortality causing a heavy burden on the healthcare system by increasing the risk of patient injury, hospitalization, prolonged length of hospital stays, readmission, and high healthcare costs (3, 4).

Early identification of hypertension in patients with CKD enables timely intervention, which may improve clinical and economic outcomes. Timely initiation of therapy enhances the opportunity to introduce kidney protective agents such as sodium-glucose cotransporter-2 (SGLT-2) inhibitors, at a stage where they may offer maximal benefit (5). Early intervention has also been associated with delayed progression to end-stage kidney disease (ESKD), reduced cardiovascular risk, and avoidance of the substantial costs associated with dialysis (6, 7). Therefore, addressing hypertension in CKD and implementing systematic screening strategies are essential to facilitate prompt diagnosis and appropriate management (8).

Isolated systolic hypertension (ISH) characterizes most CKD patients with uncontrolled blood pressure, particularly the elderly due to aortic stiffness (9–11). In addition to aging, risk factors for ISH include stage 3 or 4 CKD, diabetes mellitus, and obesity (12). Since ISH prevalence increases with age as does CVD events, the latter is driven by systolic rather than diastolic blood pressure (13). Additionally, CKD progression is governed by systolic more than diastolic blood pressure. In the Kidney Early Evaluation Program (KEEP) study, the risk of CKD progression to ESRD was not enhanced with aging when systolic blood pressure was adjusted (14). The management of ISH is in line with that of the combined systolic-diastolic hypertension (15). Additionally, resistant hypertension characterizes many CKD patients with uncontrolled blood pressure (16). This enhances both CVD and CKD progression to ESRD (17). This review aims to provide updates on current and emerging treatment therapies for the management of hypertension in CKD.

## 2 Pathophysiology of hypertension in the context of chronic kidney disease

Hypertension and CKD are strongly interrelated. The decline in kidney function causes an elevation in blood pressure, and hypertension expedites CKD progression (18). The interaction between the two disease states is complex such that, in certain situations, it is difficult to determine which disease state precedes the other. They share common risk factors, which include older age, high baseline blood pressure, obesity, diabetes mellitus, vascular atherosclerosis, obstructive sleep apnea, ethnic minorities, smoking, heavy alcohol consumption, and excessive dietary salt ingestion (19). Several factors play a role in the pathophysiology of hypertension in CKD, including renin-angiotensin-aldosterone system (RAAS) activation, imbalance in prostaglandins (PGs) or kinins, as well as nitric oxide reduction (20).

RAAS activation is well-established in CKD. Reduced kidney blood flow and glomerular filtration rate (GFR) lead to excitement of the stretch receptors of the arteriolar artery and dense plaques (the juxtaglomerular apparatus), renin secretion is subsequently increased, and RAAS is ultimately activated. RAAS activation leads to an increment in angiotensin II synthesis. Additionally, albuminuria increases angiotensin II synthesis in tubular cells in a nuclear factor (NF)-κB-dependent manner. Angiotensin II is a known potent vasoconstrictor that increases blood pressure. It also stimulates the adrenal cortex to release aldosterone, which causes kidney sodium retention, increasing blood volume. Furthermore, angiotensin II plays a pivotal role in chronic kidney hypoxia via structural and functional mechanisms (21–23).

Prostacyclin (PGI2) and PGE2 are the most important kidney PGs. PGI2 is primarily synthesized by kidney vessels and glomeruli, whereas PGE2 is primarily synthesized by the medulla. They enhance kidney blood flow and GFR in settings of decreased circulating volume. Additionally, PGI2 enhances potassium secretion by stimulating RAAS activation and ultimately aldosterone release. Similarly, PGE2 is involved in the regulation of sodium and water retention. It, via inhibition of the Na^+^-K^+^-2Cl^−^ cotransporter type 2 (NKCC2), reduces sodium reabsorption at the thick ascending limb of the loop of Henle. Furthermore, kinins, released by arginine vasopressin, augment PGE2 synthesis in the collecting duct. PGI2 and PGE2 are potent vasodilators. The decrement of their levels in the setting of CKD plays a part in hypertension development (24–26).

Nitric oxide plays several important roles in the kidneys, including hemodynamics regulation as well as promotion of natriuresis and diuresis. Its synthesis in CKD is reduced due to endothelial dysfunction. It serves as a potent vasodilator by activating soluble guanylate cyclase (sGC) in the vascular smooth muscle and causing vasodilation; thus, its reduced levels in CKD contribute to hypertension development (27–29).

## 3 Recommended blood pressure targets in patients with chronic kidney disease

The management of blood pressure in CKD is guided by several international recommendations, which differ in their proposed target thresholds. For instance, the European Society of Cardiology (ESC) 2024 guidelines and the American Heart Association/American College of Cardiology (AHA/ACC) 2025 guidelines recommend a target of <130/80 mmHg, whereas the Kidney Disease: Improving Global Outcomes (KDIGO) 2021 guidelines recommend a target of <120/80 mmHg for all patient populations, except kidney transplant recipients where the target is <130/80 mmHg (15, 30, 31). Table 1 presents a comparative overview of major international guideline recommendations for blood pressure management in patients with CKD.

**Table 1:** Comparison of hypertension management guidelines in CKD (15, 30, 31).

Recommendation category	AHA/ACC 2025	KDIGO 2021	ESC 2024
Blood Pressure Target	Systolic BP <130 mmHg in adults with CKD	Systolic BP <120 mmHg using standardized office BP if tolerated	Systolic BP of 120–129 mmHg in most adults with CKD, if tolerated
Recommended Blood Pressure Measurement Technique	Use validated and standardized BP measurement; confirm diagnosis with out-of-office measurements (HBPM or ABPM)	Recommend standardized office BP; prefer AOBP (attended/unattended); supplement with ABPM or HBPM	Prefer ABPM or HBPM; if not feasible, repeated standardized office BP measurement
CKD with Albuminuria	ACE inhibitor or ARB recommended in patients with CKD and albuminuria (eGFR <60 ml/min/1.73 m^2^, ACR ≥ 30 mg/g)	Recommend ACE inhibitor or ARB if albuminuria (≥300 mg/day). Suggest ACE inhibitor or ARB if 30–299 mg/day	ACE inhibitor or ARB recommended for all patients with albuminuria, especially when albuminuria ≥300 mg/day
CKD without Albuminuria	No specific drug class preferred	Reasonable to consider ACE inhibitor/ARB; not mandatory. However, individualize treatment	Individualized treatment based on eGFR and comorbidities; combination often required

ACE, angiotensin-converting enzyme; ARB, angiotensin II receptor blocker; AOBP, automated office blood pressure; ABPM, ambulatory blood pressure monitoring; HBPM, home blood pressure monitoring; BP, blood pressure; CKD, chronic kidney disease; eGFR, estimated glomerular filtration rate.

Current practice increasingly adopts stricter targets when tolerable. This is based on positive findings of patient-important outcomes, including mortality, from large randomized-controlled trials (RCTs) and systematic reviews and meta-analyses. For example, the Systolic Blood Pressure Intervention (SPRINT) trial randomly assigned around 10,000 patients with a systolic blood pressure of 130 mmHg or higher and an increased cardiovascular risk without diabetes to a target of less than 120 mmHg (intensive arm) or a target of less 140 mmHg (standard arm). The investigators included a subgroup of 2,646 CKD patients. The primary composite outcome was MI, other acute coronary syndromes, stroke, heart failure, or death from cardiovascular causes, and the secondary outcomes were the individual components of the primary composite outcome, death from any cause, and the composite of the primary outcome or death from any cause. Unlike the entire population of the study, the CKD subgroup did not show a statistically significant difference in the primary outcome, possibly due to being underpowered. However, the intensive arm had a significantly lower mortality rate compared with the standard arm (1.6% vs. 2.2% annual mortality rate) (32, 33).

Several systematic reviews and meta-analyses reported positive findings with stricter targets (34–38). For instance, a systematic review and meta-analysis that combined data from the Modification of Diet in Renal Disease (MDRD) trial and the African American Study of Kidney Disease and Hypertension (AASK) trial with more than one decade of follow-up. The study reported a relative risk (RR) for ESRD and mortality of 0.88 (95% CI, 0.78 to 1.00) and 0.87 (95% CI, 0.76 to 0.99), respectively. Notably, the reduction in CKD progression to ESRD was confined to those with albiminurea (36). Additionally, the posttrial follow-up of a systematic review and meta-analysis that included nine trials with 8,127 patients demonstrated a lower risk of CKD progression to ESRD (RR, 0.91; 95% CI, 0.85 to 0.99). Interestingly, when the analysis was strictly restricted to those without diabetes mellitus, a mortality benefit was demonstrated (RR, 0.78; 95% CI, 0.61 to 0.99) (37). Despite that, it is crucial to be aware that such stricter targets should be considered only when therapy tolerability is provided. Essentially, the standard of care should incorporate risk versus benefit ratio and have individualized blood pressure targets, aiming for more lenient targets in patients with symptomatic postural hypotension or very limited life expectancy. Additionally, benefits behind such stricter targets are less certain in specific populations, including those with stage 4 and stage 5 CKD, concomitant diabetes mellitus, systolic blood pressure of 120–129 mmHg, very low diastolic blood pressure, e.g., <50 mmHg, white coat hypertension, severe hypertension, as well as older or younger aged individuals (31).

Per KDIGO guidelines, standardized office blood pressure measurement is preferred to routine office blood pressure measurement, and out-of-office blood pressure measurements with home blood pressure monitoring (HBPM) or ambulatory blood pressure monitoring (ABPM) should be utilized to complement standardized office blood pressure measurement (31). The two modalities, i.e., office and out-of-office blood pressure measurements, help eliminate the chances of having white-coat or masked hypertension (31). Compared with office blood pressure measurement, out-of-office blood pressure measurement is more closely associated with CVD and end-organ damage (39–42). For instance, a systematic review and meta-analysis by Bliziotis and colleagues compared HBPM versus ABPM and /or office blood pressure measurement in predicting end-organ damage. The included studies that assessed echocardiographic left ventricular mass index (LVMI) showed a similar correlation with HBPM (coefficients r = 0.46/0.28, systolic/diastolic) as with ABPM (0.37/0.26, *P* = not significant for difference versus HBPM) and superior to office measurements (r = 0.23/0.19, *p* < 0.001/0.009 for difference versus HBPM) (41). Although ABPM has been considered the reference standard for out-of-office blood pressure measurements, HBPM confers various advantages over ABPM, including being a better measure of basal blood pressure, being widely available to patients and clinicians, as well as the availability of evidence that supports its role in better controlling office blood pressure (43). Recent evidence highlights the potential role of community pharmacy-measured blood pressure (CPBP) as a convenient and accessible alternative to ABPM (44). CPBP measurements were not significantly different from awake ABPM readings (128.0 ± 20.2 mmHg vs. 129.1 ± 15.8 mmHg; *p* = 0.8409), suggesting that community pharmacy settings may provide a viable setting for reliable blood pressure monitoring (44).

Physiologically, relative to daytime blood pressure, nocturnal blood pressure is anticipated to decrease by 10–20%. In fact, nocturnal dipping is classified into four patterns: inverse dipper (nocturnal increase in blood pressure), non-dipper (nocturnal reduction in blood pressure by <10%), normal dipping (nocturnal reduction in blood pressure by >10% and ≤20%), and extreme dipping (nocturnal reduction in blood pressure by >20%) (15). Nevertheless, some CKD patients, particularly those at later stages, do not experience such a physiological process, which adds to the existing challenges in managing and monitoring hypertension in this patient population (45–47).

## 4 Non-pharmacologic strategies for blood pressure control in chronic kidney disease

### 4.1 Sodium restriction

As salt excretion in CKD is reduced, a sodium intake of <2 g of sodium per day (or <90 mmol of sodium per day, or <5 g of sodium chloride per day) is suggested (31). Higher sodium intake has been linked to kidney damage. For instance, a systematic review by Smyth et al. included seven studies and assessed the association between sodium intake and kidney function (48). Four of the included studies were conducted on CKD patients. The study reported that high sodium intake (>4.6 g per day) accelerates kidney damage and is associated with negative kidney outcomes, including albuminuria, a greater decline in creatinine clearance, as well as a higher risk for CKD progression to ESRD (49–52). Additionally, another systematic review and meta-analysis by McMahon and colleagues included eight RCTs on CKD patients and noted that salt restriction is directly proportional to lowering systolic blood pressure (8 studies, 258 participants: MD -8.75 mmHg, 95% CI −11.33 to −6.16; I^2^ = 0%), diastolic blood pressure (8 studies, 258 participants: MD −3.70 mmHg, 95% CI -5.09 to −2.30; I^2^ = 0%) as well as albuminuria (53). Moreover, a low sodium intake is cardioprotective, and this is particularly important in CKD patients given that they are at increased risk of CVD (54). Furthermore, KIDGO guidelines suggest that caution should be experienced in following the Dietary Approaches to Stop Hypertension (DASH) diet or using potassium-containing salt substitutes, especially in advanced CKD, hyperkalemia from other causes, and hyporeninemic hypoaldosteronism (31).

### 4.2 Physical activity

Moderate-intensity physical activity for at least 150 min per week (≥30 min, 5–7 days/week) is suggested (31). Alternatively, when tolerable, vigorous-intensity physical activity for at least 75 min per week over 3 days may be performed (15, 31). Although fewer patients, when compared with non-CKD-patients, can tolerate 300 min of moderate-intensity or 150 min of vigorous-intensity physical activity per week, additional benefits can be obtained from such longer sessions (55). Various factors should be taken into consideration when recommending the form and intensity of physical activity, including the risk of falls, cognitive function, as well as cardiorespiratory fitness status (31). Although of low quality, existing evidence has demonstrated that physical activity is associated with a reduction in blood pressure, an increase in quality of life, as well as a reduction in body weight (56, 57). For example, a systematic review and meta-analysis by Heiwe and Jacobson noted that physical activity is associated with improved aerobic capacity, muscular functioning, cardiovascular function, walking capacity, and health-related quality of life, with most data being on aerobic exercise among ESRD patients (56).

### 4.3 Other lifestyle modifications

Although other lifestyle interventions, including smoking cessation, moderation in coffee and soft drinks intake, and weight reduction, are supported by well-established evidence in the general population, insufficient data are available on their risks and benefits in hypertension management among CKD patients. Nonetheless, their incorporation into the lifestyle can be reasonably considered when deemed safe and without side effects (15, 31, 58–60).

## 5 Pharmacologic management of hypertension in chronic kidney disease

### 5.1 Renin-angiotensin-aldosterone system blockade (ACEIs, ARBs, and ARNI)

Angiotensin-converting enzyme (ACE) inhibitors or angiotensin receptor blockers (ARBs) are recommended as first-line agents for patients with moderate-to-severe albuminuria, defined as an albumin-to-creatinine ratio (ACR) ≥30 mg/g (61). ACE inhibitors or ARBs are particularly preferred in the setting of severe albuminuria (ACR > 300 mg/g), as their use has been associated with improvements in patient-important outcomes, including a reduction in CKD progression to ESRD (62–64). In the Angiotensin-Converting-Enzyme Inhibition in Progressive Renal Insufficiency (AIPRI) trial, a three-year multicenter RCT, 583 patients without diabetes mellitus and with baseline proteinuria (mean urinary protein excretion of 1.8 g/day) were randomly assigned to receive benazepril or placebo. The primary endpoint was doubling baseline serum creatinine or the need for dialysis. Benazepril was associated with a significant reduction in this composite endpoint (31 vs. 57 patients; *p* < 0.001) (64). The kidney-protective effects of ACE inhibitors and ARBs also extend to patients with diabetes mellitus, particularly those with moderate-to-severe albuminuria (65–67). In the Microalbuminuria, Cardiovascular and Renal Outcomes–Heart Outcomes Prevention Evaluation (Micro-HOPE) trial, 1,140 patients with diabetes mellitus and moderate albuminuria were randomly assigned to receive ramipril or placebo. There was a RR reduction (RRR) of 28.6% (HR: 0.71; 95% CI: 0.6 to 0.9) in a composite outcome of MI, stroke, and cardiovascular death (67). With ACE inhibitors and ARBs, the rate of reduction in albuminuria is dose-proportional, i.e., the higher the dose the higher the reduction in albuminuria; hence, the maximal tolerated dose should be given (68). Evidence from landmark clinical trials consistently demonstrates the kidney-protective effects of ACE inhibitors and ARBs in patients with CKD and albuminuria, both with and without diabetes (62, 63, 65, 66). Therefore, major international guidelines recommend ACE inhibitors or ARBs as foundational therapy, particularly in patients with CKD and albuminuria, to delay disease progression and improve long-term renal outcomes (30, 31, 69, 70).

Angiotensin Receptor-Neprilysin Inhibitors (ARNIs) represent an important advancement in cardiovascular therapy by providing a dual mechanism of action through inhibiting neprilysin and RAAS. Sacubitril/valsartan, the first-in-class ARNI, is a fixed-dose combination that combines valsartan, an ARB, with sacubitril, a neprilysin inhibitor, in a 1:1 ratio within a single formulation (71). Diuretic insufficiency is a complication of CKD with hypertension, and the enhanced natriuretic peptide-mediated sodium diuresis induced by sacubitril/valsartan alleviates fluid overload, thereby lowering stroke volume and producing a more substantial antihypertensive effect (72).

A post-hoc analysis of the PARADIGM-HF trial, which enrolled 8,339 patients with heart failure with reduced ejection fraction (HFrEF), reported a mean eGFR of 68 ± 20 mL/min/1.73 m^2^, with 33% of patients having an eGFR <60 mL/min/1.73 m^2^ (73). The study compared the effects of sacubitril/valsartan and enalapril on kidney outcomes, including annual eGFR decline, uACR, a > 50% reduction in eGFR, or progression to ESRD (73). Regardless of baseline CKD status, sacubitril/valsartan was associated with a significantly slower annual decline in eGFR compared with enalapril (−1.61 vs. −2.04 mL/min/1.73 m^2^ per year; *p* < 0.001). Furthermore, sacubitril/valsartan significantly reduced eGFR decline ≥50% or progression to ESRD by 37% compared to enalapril (95% CI, 0.42–0.95; *p* = 0.028). In contrast, sacubitril/valsartan group experienced a greater increase in uACR compared enalapril group (1.20 mg/mmol [95% CI, 1.04–1.36] vs. 0.90 mg/mmol [95% CI, 0.77–1.03]; *p* < 0.001).

Similarly, a post-hoc analysis of the PARAGON-HF trial enrolled 4,822 patients with heart failure with preserved ejection fraction (HFpEF) (74). The mean baseline eGFR was 63 ± 19 mL/min/1.73 m^2^, and 47% of patients had an eGFR <60 mL/min/1.73 m^2^. The study evaluated the effect of sacubitril/valsartan versus valsartan on renal outcomes. Sacubitril/valsartan slowed the annual rate of eGFR decline compared with valsartan (−2.0 vs. −2.7 mL/min/1.73 m^2^ per year; *p* < 0.001). In addition, Sacubitril/valsartan reduced the renal composite endpoint—defined as ≥50% decline in eGFR, progression to ESRD, or renal death—compared with valsartan (1.4% vs. 2.7%; HR 0.50; 95% CI, 0.33–0.77; *p* = 0.001). Unlike the PARADIGM-HF analysis, however, uACR was not assessed in the PARAGON-HF analysis, which limits the evaluation of sacubitril/valsartan in patients with HFpEF.

A recent systematic review and meta-analysis of 14 randomized controlled trials that enrolled patients across both HFrEF and HFpEF populations (mean baseline eGFR 35 to 70 mL/min/1.73 m^2^), sacubitril/valsartan significantly reduced the risk of kidney impairment, defined as an increase in serum creatinine ≥0.3 mg/dL by 31% (OR 0.69; 95% CI, 0.59–0.80; *p* < 0.00001), and lowered the risk of ≥50% decline in eGFR or progression to ESRD by 37% (OR 0.63; 95% CI, 0.49–0.80; *p* = 0.0002) (75). Regarding albuminuria, only four studies reported uACR outcomes with inconsistent findings; some showed greater increases in uACR with sacubitril/valsartan compared with RAAS inhibitors, while others demonstrated no significant difference (75). Overall, these results suggest that ARNI therapy provides renal protection primarily by decreasing the risk of eGFR decline, whereas its effects on albuminuria remain uncertain (75).

### 5.2 Calcium channel blockers and diuretics

Given the complexity of hypertension in CKD, monotherapy is often insufficient to achieve target blood pressure levels (6). In such cases, calcium channel blockers (CCBs) and thiazide or thiazide-like diuretics are recommended as effective adjunctive therapies in the pharmacological management of hypertension in CKD (15, 30, 31). The Antihypertensive and Lipid-Lowering Treatment to Prevent Heart Attack Trial (ALLHAT) aimed to determine whether a CCB (amlodipine) or an ACE inhibitor (lisinopril) lowers coronary heart disease (CHD) or other cardiovascular events compared with a thiazide-like diuretic (chlorthalidone). The trial enrolled participants with generally preserved kidney function (mean baseline serum creatinine: 77.83 μmol/L) and did not assess albuminuria as part of the eligibility criteria. Compared with lisinopril, chlorthalidone significantly reduced the incidence of stroke, particularly among Black individuals (5.6% vs. 6.3%; RR, 1.15; 95% CI, 1.02 to 1.30; *p* = 0.02), as well as the composite outcome of CHD, stroke, treated angina without hospitalization, heart failure, or peripheral artery disease (30.9% vs. 33.3%; RR, 1.10; 95% CI, 1.05 to 1.16; *p* < 0.001) (76). Subgroup analyses from the ALLHAT trial, which evaluated renal outcomes in high-risk hypertensive patients with reduced kidney function (baseline eGFR <60 mL/min/1.73 m^2^), showed no significant differences over a 6-year period in the incidence of ESRD or the composite renal outcome across the amlodipine, lisinopril, and chlorthalidone groups (77). These findings suggest that, in patients with CKD without albuminuria, any of the standard first-line classes can be considered effective for blood pressure control based on the patient’s comorbidities, race, and treatment tolerance (30, 70).

The ACCOMPLISH trial’s prespecified secondary analysis of 2,170 patients with hypertension and CKD (baseline eGFR <60 mL/min/1.73 m^2^) demonstrated that the combination of benazepril and amlodipine significantly reduced CKD progression compared with benazepril and hydrochlorothiazide. After a mean follow-up of 2.9 years, the composite renal outcome—defined as doubling of serum creatinine, progression to ESRD, or death—occurred in 113 patients (2.0%) in the benazepril–amlodipine group versus 215 patients (3.7%) in the benazepril-hydrochlorothiazide group (HR, 0.52; 95% CI, 0.41 to 0.65; *p* < 0.0001) (78). Relatively recently, the Chlorthalidone for Hypertension in Advanced Chronic Kidney Disease (CLICK) trial provided additional insights into the efficacy of thiazide-like diuretics in patients with advanced CKD. This randomized, double-blind, placebo-controlled trial enrolled 160 adults with stage 4 CKD (mean eGFR 23.2 ± 4.1 mL/min/1.73 m^2^) and poorly controlled hypertension despite treatment with at least two antihypertensive agents. Over 12 weeks, participants receiving chlorthalidone (starting at 12.5 mg/day and titrated up to 50 mg/day) achieved a significantly greater reduction in 24-h systolic blood pressure compared to placebo, with a between-group difference of −10.5 mmHg (95% CI, −14.6 to −6.4; *p* < 0.001) (79). Secondary outcomes also favored chlorthalidone, including a greater reduction in the urinary albumin-to-creatinine ratio (uACR) compared to placebo (median percent change: −50 percentage points; 95% CI, 37 to 60; *p* < 0.001), suggesting potential cardiovascular and renal benefits beyond blood pressure reduction. More recent evidence from the Diuretic Comparison Project (DCP), a large, pragmatic, randomized trial of over 13,000 older adults with hypertension, demonstrated no significant difference between chlorthalidone and hydrochlorothiazide in preventing major adverse cardiovascular events or non–cancer-related death (HR, 1.04; 95% CI, 0.94 to 1.16; *p* = 0.45) (80). A prespecified secondary analysis focused on kidney-related outcomes and found no significant difference between the two agents in the risk of kidney disease progression, including incident CKD, doubling of serum creatinine, or need for dialysis. Furthermore, the incidence of adverse effects such as hypokalemia was higher in the chlorthalidone group (81). These findings challenge earlier beliefs about the superiority of chlorthalidone and support the notion that both agents may be similarly effective and safe for blood pressure control, even in patients at risk of developing CKD.

### 5.3 Sodium-glucose cotransporter-2 inhibitors, nonsteroidal mineralocorticoid receptor antagonist, and incretin-based therapies

Sodium-glucose cotransporter-2 (SGLT-2) inhibitors have emerged as an important therapeutic class in patients with CKD. Although their antihypertensive effect is modest, SGLT-2 inhibitors are believed to lower blood pressure primarily through osmotic diuresis, natriuresis, and inhibition of proximal tubular sodium reabsorption via blockade of the sodium–hydrogen exchanger 3 (NHE3) (82). These mechanisms lead to plasma volume reduction and improved vascular function, further reinforcing their overall cardio-renal protective profile in patients with CKD (82). Robust evidence from landmark clinical trials and real-world data has demonstrated that SGLT-2 inhibitors significantly slow the progression of CKD, reduce albuminuria, and lower the risk of cardiovascular events (20, 83, 84). Subgroup analyses from a recent meta-analysis confirmed that the cardiovascular and kidney protective effects of SGLT-2 inhibitors were consistently observed in patients with CKD and an eGFR ≤60 mL/min/1.73 m^2^ (85). These cardiovascular and kidney benefits extend to patients with CKD stage 4, as SGLT-2 inhibitors were associated with a significant reduction in cardiovascular events (RR, 0.76; 95% CI, 0.54 to 0.82), hospitalization for heart failure (RR, 0.74; 95% CI, 0.55 to 1.00), and renal composite outcomes (RR, 0.78; 95% CI, 0.68 to 0.88) (85). Given the frequent coexistence of hypertension, heart failure, and type 2 diabetes in patients with CKD, the addition of SGLT-2 inhibitors offers a multifaceted therapeutic strategy that includes modest blood pressure-lowering effects (86). SGLT-2 inhibitors are recommended for initiation in patients with an eGFR ≥20 mL/min/1.73 m^2^ and, once started, may be continued even if kidney function declines below this threshold (86).

Recent therapeutic advances that offer modest blood pressure reduction and cardiovascular and kidney protection include incretin therapies and nonsteroidal mineralocorticoid receptor antagonist (MRA) finerenone (87–91). Current clinical guidelines recommend the use of Glucagon-Like Peptide-1 (GLP-1) receptor agonists and SGLT-2 inhibitors in adults with type 2 diabetes and CKD to reduce cardiovascular risk and slow the progression of kidney disease, irrespective of glycemic control (92). In a real-world cohort of 44,415 adults with type 2 diabetes and CKD (8,783 receiving combination therapy and 35,532 receiving SGLT-2 inhibitor monotherapy), combination treatment with GLP-1 receptor agonists and SGLT-2 inhibitors resulted in significantly smaller declines in kidney function (93). At 12 and 18 months, the adjusted mean differences in eGFR were 1.17 mL/min/1.73 m^2^ (95% CI, 0.22 to 2.13; *p* = 0.016) and 1.09 mL/min/1.73 m^2^ (95% CI, 0.03 to 2.15; *p* = 0.043), respectively (93). These findings suggest that GLP-1 receptor agonists may confer incremental kidney protection when used in combination with SGLT-2 inhibitors. Recent modeling analysis estimated that combination therapy with an SGLT-2 inhibitor, a GLP-1 receptor agonist, and finerenone in patients with type 2 diabetes and albuminuria could reduce the risk of major adverse cardiovascular events by 35% (HR, 0.65; 95% CI, 0.55 to 0.76), hospitalization for heart failure by 55% (HR, 0.45; 95% CI, 0.34 to 0.58), and CKD progression by 36% (HR, 0.42; 95% CI, 0.51 to 0.80), compared with conventional care (94). The incremental kidney benefits associated with the addition of finerenone are based on simulation modeling and should be interpreted with caution in the absence of supporting randomized trials or real-world data.

Although the primary benefits of finerenone and incretin therapies relate to kidney and cardiovascular outcomes, clinical data also support their modest blood pressure-lowering effects. In a substudy of the Mineralocorticoid Receptor Antagonist Tolerability Study–Diabetic Nephropathy (ARTS-DN) phase 2b trial, 240 patients with type 2 diabetes and CKD underwent 24-h ABPM. Finerenone at doses of 10–20 mg once daily significantly reduced systolic blood pressure compared with placebo, with placebo-adjusted reductions at Day 90 ranging from −8.3 mmHg (95% CI, −16.6 to 0.1) to −11.2 mmHg (95% CI, −18.8 to −3.6), based on the dose administered (95). By comparison, a meta-analysis of randomized controlled trials that evaluated SGLT-2 inhibitors reported a placebo-adjusted reduction in 24-h systolic blood pressure of −3.62 mmHg (95% CI -4.29 to −2.94). Although direct head-to-head comparisons are lacking, the available data suggest that finerenone may exert a comparatively greater antihypertensive effect in patients with type 2 diabetes and CKD (96).

Incretin therapies exert their effects through activation of GLP-1 and GIP (Glucose-dependent Insulinotropic Polypeptide) receptors expressed in multiple tissues, including the kidneys, vasculature, and central nervous system (97). This broad receptor distribution underlies their multifaceted physiological actions, including modest reductions in blood pressure (97). The antihypertensive effect is thought to result from a combination of enhanced natriuresis, reduced arterial stiffness, and improved endothelial function (98). Additionally, these agents may contribute to blood pressure-lowering effects indirectly through weight loss (99). Agents associated with greater weight loss tend to exert a more pronounced effect on blood pressure. This relationship was supported by findings from the SURMOUNT-5 trial, which evaluated once-weekly subcutaneous tirzepatide (10 or 15 mg) versus semaglutide (1.7 or 2.4 mg) over 72 weeks in adults with obesity and without diabetes (100). Tirzepatide resulted in a greater reduction in systolic blood pressure compared to semaglutide (−10.2 mmHg vs. -7.7 mmHg), with a between-group difference of −2.5 mmHg (95% CI, −3.9 to −1.1) (100). A meta-analysis of RCTs demonstrated that each kilogram of weight loss is associated with an approximate reduction of 1 mmHg in systolic blood pressure (−1.05 mmHg; 95% CI, −1.43 to −0.66) (101). Although no RCT has specifically evaluated blood pressure reduction of incretin therapies as a primary endpoint, some studies offer insight into the potential antihypertensive effects. For instance, the FLOW trial, which assessed the renal outcomes of semaglutide in 3,533 patients with type 2 diabetes and CKD, reported a significant reduction in systolic blood pressure with semaglutide compared to placebo (−2.23 mmHg; 95% CI, −3.33 to −1.13) over 104 weeks (88). Nevertheless, the clinical impact of incretin therapies may differ depending on the specific agent and individual patient characteristics. Variability in weight loss across agents may influence their antihypertensive effects, and the heterogeneity of comorbidities in patients with CKD highlights the need for individualized treatment decisions.

Despite their modest antihypertensive effects, SGLT-2 inhibitors, incretin therapies, and finerenone offer substantial ancillary benefits that extend beyond blood pressure control. Findings from a large-scale meta-analysis involving approximately 350,000 participants demonstrated that a 5 mmHg reduction in systolic blood pressure was associated with an approximate 10% reduction in the risk of major adverse cardiovascular events (102). Additionally, these agents exert anti-inflammatory, antifibrotic, and metabolic effects, which contribute to improved cardiovascular and kidney outcomes (98, 103, 104). Their ability to target multiple pathophysiological pathways makes them valuable components of an integrated, patient-centered approach to managing hypertension in CKD. Nonetheless, considerations such as safety, cost, accessibility, and the magnitude of blood pressure reduction required should guide clinical decision-making. Additionally, given the multifactorial nature of resistant hypertension in CKD, many patients ultimately require combination therapy involving multiple first- and second-line antihypertensive agents, such as beta-blockers, alpha-blockers, central sympatholytics, or direct vasodilators, to achieve adequate blood pressure control.

## 6 Special populations

### 6.1 Elderly patients with CKD

According to the KDIGO 2021 guideline on blood pressure management in CKD, a target systolic BP of less than 120 mmHg is recommended when measured with standardized office techniques (31). Nonetheless, a more conservative BP-lowering approach should be considered in elderly patients (31). The SPRINT trial included 9,361 patients with cardiovascular risk, of whom more than 40% of patients with CKD were ≥75 years. In subgroup analysis these patients, intensive BP control (target SBP < 120 mmHg) compared with standard control (<140 mmHg) reduced the primary composite cardiovascular outcome by 34% (HR 0.66; 95% CI, 0.51 to 0.85) and a 33% reduction in all-cause mortality (HR 0.67; 95% CI, 0.49 to 0.91) (32). Similarly, the STEP trial further included 8,511 patients aged 60–80 years, with a subgroup of pre-existing CKD, intensive SBP group (110–130 mmHg) reduced the primary cardiovascular composite outcome by 26% compared to the standard SBP group (130–150 mmHg) (HR 0.74; 95% CI, 0.60 to 0.92; *p* = 0.007) (105). Importantly, the average achieved SBP in both SPRINT and STEP intensive groups was 121–130 mmHg, Nevertheless, some studies have reported that targeting systolic BP below 120 mmHg in elderly patients may be associated with mortality (106). In regard to the preferred drug class for this population, the KDIGO guideline does not recommend a specific mention of age-based selection (31).

### 6.2 Kidney transplant recipients

In kidney transplant recipients, dihydropyridine CCBs are often the preferred initial agents for managing hypertension (31). This preference is based on their ability to counteract calcineurin inhibitor–induced vasoconstriction and their neutral or favorable effects on graft perfusion. Dihydropyridine CCB use was associated with a mean reduction in graft loss of 38% (RR: 0.62; 95% CI: 0.43 to 0.90) in a meta-analysis included 926 kidney transplant recipients (107). In comparison, same analysis showed that ARBs were associated with a mean reduction in graft loss of 65% (RR: 0.35; 95% CI: 0.15 to 0.84) (31). The outcome of preventing graft loss is critical and among the top valued outcomes among kidney transplant recipients (108). ACE inhibitors or ARBs should be also considered in the presence of albuminuria or compelling cardiovascular indications (30, 31, 70). However, their use early post-transplant should be approached with caution due to the potential risk of hyperkalemia and acute declines in eGFR (31).

## 7 Novel antihypertensive therapies relevant to high-risk populations

Several antihypertensive agents with novel mechanisms of action are in development and may offer therapeutic advantages for resistant hypertension across diverse patient populations, including those with CKD. Spironolactone is commonly recommended as add-on therapy for resistant hypertension (30, 109). However, its use is limited in patients with CKD due to the increased risk of hyperkalemia (30, 109). Endothelin-receptor antagonists (ERAs) have been explored for resistant hypertension since the late 1990s (110). Although early trials with bosentan and darusentan demonstrated blood pressure-lowering effects, further development was stopped after phase 3 studies failed to show consistent efficacy in office-based blood pressure measurements (111–113). Aprocitentan, a dual endothelin A and B receptor antagonist, was approved in March 2024 for the treatment of resistant hypertension after demonstrating clinically meaningful blood pressure reductions in the phase 3 PRECISION trial (113, 114). The trial enrolled 730 patients with resistant hypertension who were receiving standardized background therapy to evaluate the change in mean systolic blood pressure from baseline to week 4 as the primary endpoint (114). The study also included a secondary endpoint measuring uACR to assess renal outcomes (114). Compared with placebo, aprocitentan reduced systolic blood pressure at week 4 by a mean of 3.8 mmHg in the 12.5 mg group (97.5% CI -6.8 to −0.8; *p* = 0.0042) and 3.7 mmHg in the 25 mg group (97.5% CI -6.7 to −0.8; *p* = 0.0046) (114). Aprocitentan also produced a clinically meaningful reduction in albuminuria, with uACR decreasing by 28 and 31% in the 12.5 mg and 25 mg groups, respectively (114). The most commonly reported adverse effect was fluid retention or edema, which occurred in a dose-dependent manner in 9.1 and 18.4% of patients receiving aprocitentan 12.5 mg and 25 mg, respectively, compared with 2.1% in the placebo group. Given the high prevalence of volume-related complications in CKD, this adverse effect may be particularly important to consider when selecting therapy in this population. This adverse effect is thought to result from endothelin A and B receptor blockade, which may lead to vasodilation, activation of vasopressin and aldosterone pathways, and increased vascular permeability (114, 115).

Ocedurenone, a nonsteroidal MRA, has shown potential in reducing blood pressure among patients with stage 3–4 CKD, with a lower observed incidence of hyperkalemia compared to traditional steroidal MRAs such as spironolactone (116). The BLOCK-CKD trial, a phase 2b, randomized, double-blind, placebo-controlled study, evaluated its efficacy and safety in 162 patients with stage 3b or 4 CKD and uncontrolled hypertension (116). Participants were randomized to receive placebo or ocedurenone at 0.25 mg or 0.5 mg daily for 84 days. The primary endpoint—change in trough cuff seated systolic blood pressure—was met, with placebo-adjusted reductions of −7.0 mmHg (95% CI: −13.7 to −0.3; *p* = 0.0399) and −10.2 mmHg (95% CI: −16.7 to −3.6; *p* = 0.0026) for the 0.25 mg and 0.5 mg groups, respectively (116). Rates of hyperkalemia were low and comparable between groups (116). However, the subsequent phase 3 CLARION-CKD trial was terminated early after an interim analysis concluded that the study was unlikely to meet its primary efficacy endpoint (117).

Another investigational strategy in resistant hypertension involves the selective inhibition of aldosterone synthase. Baxdrostat, a first-in-class, highly selective aldosterone synthase inhibitor, reduces aldosterone biosynthesis by targeting the CYP11B2 enzyme while sparing 11β-hydroxylase (CYP11B1), thereby minimizing cortisol suppression (118, 119). This selectivity offers a mechanistic advantage over MRA like spironolactone, which act downstream and are associated with broader hormonal side effects (118, 119). Baxdrostat has shown promise in lowering blood pressure in patients with treatment-resistant hypertension. In the phase 2b BrigHTN trial—a randomized, double-blind, placebo-controlled study involving 275 patients with resistant hypertension—baxdrostat significantly reduced systolic blood pressure in a dose-dependent manner (118). Compared with placebo, the 1 mg dose reduced systolic blood pressure by 8.1 mmHg (95% CI, −13.5 to −2.8; *p* = 0.003) and the 2 mg dose by 11.0 mmHg (95% CI, −16.4 to −5.5; *p* < 0.001) at week 12 (118). Notably, the trial did not evaluate renal outcomes. Two phase 3 trials—BaxHTN (NCT06034743) and Bax24 (NCT06168409)—are currently investigating baxdrostat in resistant and uncontrolled hypertension, with results expected in late 2025. Lorundrostat is another aldosterone synthase inhibitor currently under investigation for hypertension management. In a recent phase 2 randomized, double-blind, placebo-controlled trial (Advance-HTN), lorundrostat significantly reduced 24-h ambulatory systolic blood pressure in patients with uncontrolled or treatment-resistant hypertension (120). After 12 weeks of treatment, the placebo-adjusted reductions were −7.9 mmHg (97.5% CI, −13.3 to −2.6) in the stable-dose group (50 mg daily) and −6.5 mmHg (97.5% CI, −11.8 to −1.2) in the dose-adjustment group (50 to 100 mg daily), compared with placebo (120). These findings support the potential of targeting aldosterone synthesis upstream in the renin-angiotensin-aldosterone pathway as a novel approach to managing difficult-to-control hypertension.

A novel approach in antihypertensive therapy targets gene silencing mechanisms to modulate the renin–angiotensin–aldosterone system upstream. Zilebesiran is a subcutaneously administered RNA interference (RNAi) therapeutic that suppresses hepatic production of angiotensinogen, thereby lowering blood pressure by inhibiting the downstream effects of angiotensin II (121). This mechanism offers the potential for sustained blood pressure control with infrequent dosing, which could improve adherence and reduce variability associated with daily antihypertensive therapy (121). The KARDIA-3 trial (NCT06272487) is an ongoing multicenter, double-blind, randomized, placebo-controlled phase 2 study designed to evaluate the efficacy and safety of zilebesiran in patients with uncontrolled hypertension and either established cardiovascular disease, high cardiovascular risk, or advanced CKD (122). Table 2 provides a comparative summary of novel and emerging therapies for resistant hypertension.

**Table 2:** Summary of novel and investigational therapies for resistant hypertension (114, 118, 120, 122).

Medication/landmark trial	Approval date/study start	Study phase	Mode of action	Starting dose	Most common adverse events	CKD-related endpoint
Aprocitentan (PRECISION)	March 2024^*^	Phase 3	Dual endothelin receptor antagonist (ERA)	25 mg PO OD	Fluid retention, headache, and mild anemia	Albuminuria reduced by 28–31% (secondary endpoint)
Baxdrostat (BrigHTN)	October 2020	Phase 2	Aldosterone synthase inhibitor	0.5 mg PO OD	Hyperkalemia	NA
Lorundrostat (Target-HTN)	July 2021	Phase 2	Aldosterone synthase inhibitor	50 mg PO OD	Hyperkalemia	Change in eGFR was not tested for significance
Zilebesiran (KARDIA-3)	February 2024	Phase 2	Angiotensinogen synthesis inhibitor	150, 300, or 600 mg SC as a single dose	Injection-site reaction	Awaiting the results

eGFR, estimated glomerular filtration rate; NA, not applicable; OD, once daily; PO, orally; SC, subcutaneously. * Aprocitentan is the only approved medication. The other dates represent the study start date for each respective medication.

## 8 Conclusion

The management of hypertension in chronic kidney disease remains a cornerstone of mitigating cardiovascular morbidity and delaying renal disease progression. While therapeutic recommendations differ slightly across clinical practice guidelines, there is consensus regarding the foundational role of lifestyle interventions and first-line pharmacologic therapy in patients with CKD and albuminuria. ACE inhibitors and ARBs remain the first-line therapies, particularly in patients with CKD and albuminuria. However, monotherapy is frequently insufficient in the setting of hypertension in CKD; therefore, other therapies, including calcium channel blockers (CCBs) and thiazide or thiazide-like diuretics, are usually added. Additionally, SGLT-2 inhibitors, incretin therapies, and finerenone have proven to provide cardiovascular and renal benefits beyond their modest antihypertensive effects and can be considered effective adjunctive therapies. Several emerging therapies with novel mechanisms of action are being developed, which may be particularly advantageous for resistant hypertension. This review provides a novel contribution by summarizing the most recent evidence on blood pressure management in CKD, with an emphasis on integrating new therapeutic options that are underrepresented in previous literature.
